# Clinicopathological Features and First-Line Treatment Outcomes of Geriatric Patients With Extensive-Stage Small Cell Lung Cancer: A Multicenter Study

**DOI:** 10.7759/cureus.35710

**Published:** 2023-03-02

**Authors:** Aysegul İlhan, Fatih Gurler, Funda Yilmaz, Erdogan Seyran, Vural Bastug, Bugra Gorgulu, Emrah Eraslan, Özgen Ahmet Yıldırım, Ozan Yazici, Ömür Berna Çakmak Öksüzoğlu

**Affiliations:** 1 Department of Medical Oncology, University of Health Sciences, Dr. Abdurrahman Yurtarslan Ankara Oncology Training and Research Hospital, Ankara, TUR; 2 Department of Internal Medicine, Gazi University School of Medicine, Ankara, TUR; 3 Department of Medical Oncology, Gazi University, Ankara, TUR

**Keywords:** survival outcomes, systemic chemotherapy, geriatric patients, geriatric patient population, extensive stage, small-cell lung cancer

## Abstract

Introduction

The geriatric patient population diagnosed with extensive stage small cell lung cancer (SCLC) is underrepresented in clinical studies. We aimed to evaluate the clinicopathological characteristics, first-line treatment patterns and treatment outcomes of patients aged 65 years or older with extensive stage SCLC.

Material and methods

In this multicenter, retrospective cohort study, patients aged 65 years or older, diagnosed with extensive-stage SCLC, between January 2009 and December 2021 were included. Patients who were under 65 years of age at the time of diagnosis and did not develop progression after curative treatment and patients with a second malignancy were excluded from the study. The clinicopathological characteristics, first-line treatment patterns and treatment outcomes were analyzed.

Results

A total of 132 patients were included in the study. The median age was 70 years (range:65-91), and 118 (89.4%) patients were male. There were 77 (58.3%) patients with eastern cooperative oncology group (ECOG) performance status (PS) of 0-1. There were 26 (19.7%) patients in the limited stage disease and 106 (80.3%) patients in the extensive stage disease at the time of diagnosis. First-line chemotherapy was given to 86 (65.2%) patients. Of the patients who could not receive treatment, 18 patients (13.6%) due to patient refusal, and 28 patients (21.2%) due to comorbid diseases and poor performance status with organ dysfunctions. The most common treatment regimen used as first-line treatment was cisplatin+etoposide (n=47, 54.7%), and followed by carboplatin+etoposide (n=39, 45.3%). First-line chemotherapy responses were complete response in 4 (4.7%) patients, partial response in 35 (40.7%) patients, stable disease in 13 (15.1%) patients, and progressive disease in 34 (39.5%) patients. The most common grade 3-4 adverse events was neutropenia in 33 (38.4%) patients. Forty nine patients (57.0%) completed the planned first-line treatment. The mPFS was 6.1 months and the mOS was 8.2 months with first-line treatment. We found that ECOG PS status was the most important negative prognostic factor for both PFS and OS. There was no difference between carboplatin+etoposide and cisplatin+etoposide regimens in terms of PFS, OS, adverse events and treatment compliance.

Conclusion

Thus, it may be an appropriate approach not to give up chemotherapy treatment easily in elderly patients with a diagnosis of extensive stage SCLC. It should be kept in mind that finding factors that might affect the prognosis and tailoring the tretment precisely on case-by-case basis in geriatric cancer patients have an impact on survival.

## Introduction

Small cell lung cancer (SCLC) is a neuroendocrine tumor characterized by high growth rate and early metastasis development [1]. It accounts for about 12-15% of all lung cancers. It is the type of lung cancer with the strongest etiological relationship with smoking. At diagnosis, nearly 75% of cases are presented with extensive-stage disease. While the five-year survival rate is 10-13% in limited-stage disease, it is around 1-2% in the extensive stage. SCLC peaks in the seventh decade and approximately 30-40% of patients are aged 70 years or older [2-4].

Although the geriatric patient population constitutes a significant proportion of patients with SCLC, this age group is not adequately represented in clinical studies. In general, standard treatment protocols are recommended for geriatric patients with good performance status and preserved organ functions, just as in younger patients. For severe comorbid diseases or geriatric patients with low-performance status, there is no clear consensus that determines treatment [5].

This study aimed to evaluate the clinicopathological characteristics, first-line treatment patterns, and treatment outcomes of patients aged 65 years or older with extensive-stage SCLC.

## Materials and methods

Patient population and data collection

In this multicenter, retrospective cohort study, patients aged 65 years or older, diagnosed with extensive-stage SCLC between January 2009 and December 2021 in the Medical Oncology Departments of the University of Health Sciences Dr. Abdurrahman Yurtaslan Ankara Oncology Training and Research Hospital, Ankara, Türkiye, and Gazi University School Medicine, Ankara, Türkiye, were included. Patients who were under 65 years of age at the time of diagnosis and did not develop progression after curative treatment and patients with a second malignancy were excluded from the study.

The baseline demographic characteristics of the patients (gender, age at diagnosis, and smoking status), clinicopathological data (Eastern Cooperative Oncology Group (ECOG) performance status (PS)), and tumor characteristics (stage and metastasis sites), treatment characteristics (palliative chemotherapy options, number of chemotherapy cycles, and treatment responses), laboratory findings (lactate dehydrogenase-LDH), disease progression, and survival data were examined and transferred to the database. American Joint Committee on Cancer (AJCC) Cancer Staging Manual, 8th Edition, was used for disease staging.

The two chemotherapy protocols given to the patients included in the study were as follows: (i) a combination of cisplatin and etoposide (cisplatin 80 mg/m^2^/day IV on day 1, etoposide 100 mg/m^2^/day IV on days 1-3), or (ii) a combination of carboplatin and etoposide (carboplatin area under the curve 5 (AUC5) IV on day 1, etoposide 100 mg/m^2^/day IV on days 1-3). Both regimens were given up to six cycles.

Response to chemotherapy was defined according to response evaluation in solid tumors criteria 1.1 (RECIST 1.1). Complete response (CR) was defined as the disappearance of all target lesions, the short axis of all pathological lymph nodes <10 mm; partial response (PR) was defined as a reduction of at least 30% in the sum of the diameters of the target lesions; progressive disease (PD) was defined as the appearance of one or more new lesions or the size of the target lesions increasing by 20% of the sum of the long diameters; and stable disease (SD) was defined as neither sufficient reduction to be considered as PR nor sufficient increase to be considered as PD.

Statistical analysis

IBM SPSS Statistics for Windows, Version 23.0 (Released 2015; IBM Corp., Armonk, New York, United States) was used for data analysis. Progression-free survival (PFS) was defined as the time from the beginning of chemotherapy treatment to disease progression or death, and overall survival (OS) was defined as the time from the date of extensive-stage diagnosis to death. Survival analyses were performed by the Kaplan-Meier method and subgroups were compared by log-rank test. Factors that may be related to PFS and OS were investigated by univariate analysis. Factors that showed significant association with survival were evaluated by multivariate Cox regression analysis. P<0.05 was considered statistically significant.

Ethical approval

Ethical approval was obtained from the Ethics Committee of the University of Health Sciences, Dr. Abdurrahman Yurtaslan Ankara Oncology Training and Research Hospital (approval number: 2022-04/1798,20.04.2022). Our study complies with the principles of the Helsinki Declaration.

## Results

General patient characteristics

A total of 132 patients were included in the study. The median age was 70 years (range:65-91), and 118 (89.4%) patients were male. There were 77 (58.3%) patients with ECOG PS of 0-1. There were 26 (19.7%) patients in the limited stage and 106 (80.3%) patients in the extensive stage at the time of diagnosis. Sixty-five (49.2%) patients had liver metastasis and the liver was the most common site of metastasis. First-line treatment was given to 86 (65.2%) patients. Of the patients who could not receive treatment, 18 (13.6%) did not get treatment due to patient refusal, and in 28 it was due to poor performance status with organ dysfunctions. The patient and tumor characteristics are shown in Table 1.

**Table 1:** Patient characteristics of the study population (N=132) ECOG: Eastern Cooperative Oncology Group; PS: performance status

Patient Characteristics				N (%)
Median age, years (range)				70 (65-91)
Sex	Female			14 (10.6%)
	Male			118 (89.4%)
ECOG PS		0-1		77 (58.3%)
		≥2		55 (41.7%)
Smoking status	Never smoked			9 (6.9%)
	Former smoker			54 (40.9%)
	Current smoker			69 (52.3%)
Pack-years	<50 packet			55 (41.7%)
	≥50 packet			68 (51.5%)
Stage at initial diagnosis	Limited stage			26 (19.7%)
	Extensive stage			106 (80.3%)
Metastasis sites	Lung			45 (34.1%)
	Pleural			14 (10.6%)
	Liver			65 (49.2%)
	Brain			35 (26.5%)
	Bone			61 (46.2%)
	Others			21 (15.9%)
Treatment modalities (limited stage)	No treatment			1 (0.8%)
	Chest radiation and chemotherapy			23 (17.4%)
	Surgery and chemotherapy			3 (2.3%)
	Prophylactic cranial irradiation			8 (6.1%)
Treatment modalities (extensive stage)	No active treatment			46 (34.8%)
	Chemotherapy			86 (65.2%)
No systemic treatment			Patient refusal	18 (13.6%)
			Poor performance status	28 (21.2%)

Treatment features

Of the 132 patients evaluated for the study, 86 (65.1%) received first-line treatment and 24 (27.9%) received second-line treatment. The most common treatment regimen used as first-line treatment was cisplatin+etoposide chemotherapy regimen (n=47, 54.7%), followed by carboplatin+etoposide (n=39, 45.3%). First-line chemotherapy responses were CR in four (4.7%) patients, PR in 35 (40.7%) patients, SD in 13 (15.1%) patients, and PD in 34 (39.5%) patients. The most common grade 3-4 adverse event was neutropenia in 33 (38.4%) patients. Of note, neutropenic fever was observed in 10 (11.8%) patients. Forty-nine (57.0%) patients completed the planned first-line cycles. However, first-line treatment was terminated in 17 (19.8%) patients due to death, in 10 (11.6%) patients due to disease progression, and in 10 (11.6%) patients due to treatment intolerance (Table 2). Topotecan was the most frequent second-line treatment (n=13, 15.1%) (Table 2).

**Table 2:** Outcomes with first-line treatment (n=86)

Treatment Protocols				Cisplatin+etoposide	47 (54.7%)		
				Carboplatin+ etoposide	39 (45.3%)		
Median number of treatment cycles						5.5 (1-14)
Best Responses			Complete Response			4 (4.7%)
			Partial Response			35 (40.7%)
			Stable Disease			13 (15.1%)
			Progressive Disease			34 (39.5%)
Reason of Treatment Termination	Completion of the planned treatment					49 (57.0%)
	Death					17 (19.8%)
	Disease progression					10 (11.6%)
	Treatment intolerance					10 (11.6%)
Grade III-IV Adverse Events (%)		Anemia				16 (18.6%)
		Thrombocytopenia				16 (18.6%)
		Neutropenia				33 (38.4%)
		Neutropenic fever				10 (11.6%)
		Liver toxicity				2 (2.4%)
Second-line Treatment						24 (27.9%)
Second-line Treatment Protocols	Topotecan					13 (15.1%)
	Carboplatin/cisplatin+ etoposide					5 (5.8%)
	Paclitaxel					4 (4.7%)
	Other					2 (2.3%)

Survival outcomes

The median PFS (mPFS) was 6.1 months, and the median OS was 8.2 months with first-line treatment. There was no statistically significant difference regarding PFS between the subgroups formed by considering the variables such as gender, age (below or above the median age), stage at diagnosis (de novo metastatic, recurrent metastatic), smoking status, and metastasis sites. The mPFS was 7.7 (95%CI 5.9-9.6) months and six (95%CI 0.4-4.4) months for those with ECOG PS of 0-1 and ≥2, respectively (p<0.001). The mPFS was 9.8 (95%CI 6.7-12.9) months and 5.8 (95%CI 4.6-7.1) months for those with and without brain metastases, respectively (p<0.043). The mPFS was 4.3 (95%CI 2.0-6.6) months and 8.3 (95%CI 6.6-10.0) months for those with and without liver metastasis, respectively (p<0.001). The mPFS below-median and above-median lactate dehydrogenase (LDH) value measured at the time of metastatic diagnosis were 7.9 (95%CI 5.4-10.4) months and 3.8 (95%CI 1.1-6.5) months, respectively (p<0.020) (Table 3). In the multivariate analysis to estimate factors that have an effect on PFS with first-line treatment, it was observed that an ECOG PS of ≥2 negatively affected the PFS (HR = 1.80, 95%CI 1.04-3.23, p <0.036).

**Table 3:** Univariate and multivariate cox-regression analysis results including factors that may affect progression-free survival HR: hazard ratio; LDH: lactate dehydrogenase; ECOG: Eastern Cooperative Oncology Group; PS: performance status

Variable		Univariate		Multivariate	
		PFS, median, months (95%)	p-value	Hazard ratio for PFS (95% CI)	p-value
Gender	Female	6.0 (1.7-10.2)	0.949		
	Male	6.1 (5.0-7.3)			
Age	< 70-year-old	6.2 (4.8-7.5)	0.735		
	≥ 70-year-old	6.0 (4.5-7.5)			
ECOG PS	0-1	7.7 (5.9-9.6)	0.001	1.04-3.23	0.036
	≥2	2.4 (0.4-4.4)			
Stage at initial diagnosis	Limited stage	5.0 (2.6-7.4)	0.729		
	Extensive stage	6.2 (5.1-7.3)			
Smoking status	Never smoked	3.9 (0.0-9.6)	0.993		
	Former smoker	5.8 (5.3-6.3)			
	Current smoker	7.0 (4.7-9.3)			
Metastasis Site	Lung	5.7 (2.2-9.2)	0.256		
	Liver	4.3 (2.0-6.6)	0.001	0.91-2.63	0.104
	Pleura	5.7 (2.4-9.0)	0.340		
	Brain	9.8 (6.7-12.9)	0.043	0.38-1.23	0.207
	Bone	6.2 (4.6-7.7)	0.310		
Chemotherapeutic agent	Carboplatin + etoposide	7.0 (5.6-8.4)	0.763		
	Cisplatin+ etoposide	6.0 (5.3-6.8)			
LDH	<359	7.9 (5.4-10.4)	0.020	0.73-2.23	0.386
	≥359	3.8 (1.1-6.5)			

There was no statistically significant difference regarding OS between the subgroups formed by considering the variables such as gender, age (below or above median), stage at diagnosis (de novo metastatic, recurrent metastatic), smoking status ,and metastasis sites. The median OS (mOS) was 10.4 (95%CI 7.5-13.4) months and 3.3 (95%CI 1.3-5.3) months for those with ECOG PS of 0-1 and ≥2, respectively (p<0.001). The mOS was 6.1 (95%CI 1.7-10.6) and 9.4 (95%CI 6.2-12.7) months for those with and without lung metastases, respectively (p<0.044). The mOS was 5.7 (95%CI 2.6-8.9) months and 11.4 (95%CI 19.6-13.1) months for those with and without liver metastases, respectively (p<0.003). The mOS was 11.4 (95%CI 9.8-13.0) months and 3.9 (95%CI 0.0-8.4) months, in below and above the median LDH value at the time of metastatic diagnosis, respectively (Table 4). In the multivariate analysis to estimate factors those have effect on OS, it was found that an ECOG PS of ≥2 (HR = 1.90, 95%CI 1.08-3.40, p<0.026), and lung metastasis (HR = 1.80, 95%CI 1.00-3.20, p< 0.047) negatively affected the OS (Table 4).

**Table 4:** Univariate and multivariate cox-regression analysis results including factors that may affect overall survival HR: hazard ratio; LDH: lactate dehydrogenase; ECOG: Eastern Cooperative Oncology Group; PS: performance status

Variable		Univariate		Multivariate	
		Overall Survival, median, months (95% CI)	p-value	HR for Overall Survival (95% CI)	p-value
Gender	Female	8.0 (0.7-15.2)	0.366		
	Male	8.1 (5.7-10.4)			
Age	< 70-year-old	8.4 (4.8-11.9)	0.591		
	≥ 70-year-old	8.0 (6.5-9.6)			
ECOG PS	0-1	10.4 (7.5-13.4)	<0.001	1.90 (1.08-3.40)	0.026
	≥2	3.3 (1.3-5.3)			
Stage at initial diagnosis	Limited stage	5.6 (4.3-7.0)	0.956		
	Extensive stage	8.4 (6.3-10.5)			
Smoking status	Never smoked	8 (0.0-17.2)	0.859		
	Former smoker	7.9 (5.0-10.8)			
	Current smoker	8.6 (4.9-12.4)			
Metastasis Site	Lung	6.1 (1.7-10.6)	0.044	1.80 (1.00-3.20)	0.047
	Liver	5.7 (2.6-8.9)	0.003	1.30 (0.70-2.20)	0.270
	Pleura	8.0 (2.2-13.8)	0.160		
	Brain	11.7 (8.5-15.0)	0.086		
	Bone	9.4 (6.1-12.8)	0.891		
Chemotherapeutic agent	Carboplatin+etoposide	8.1 (4.7-11.4)	0.689		
	Cisplatin+ etoposide	8.4 (5.4-11.4)			
LDH	<359	11.4 (9.8-13.0)	0.007	1.60 (0.94-2.97)	0.078
	≥359	3.9 (0.0-8.4)			

Outcomes of carboplatin+etoposide and cisplatin+etoposide were also compared. The mPFS was seven months (95%CI 5.6-8.4) in the carboplatin+etoposide group and six months (95%CI 5.3-6.8) in the cisplatin+etoposide group, and the difference was not statistically significant (p = 0.763) (Table 3). The mOS was 8.1 months (95%CI 4.7-11.4) in the carboplatin+etoposide group and 8.4 months (95%CI 5.4-11.4) in the cisplatin+etoposide group, and the difference was not statistically significant (p = 0.689) (Table 4). There was no difference between carboplatin+etoposide and cisplatin+etoposide groups regarding adverse events and treatment compliance (Table 5).

**Table 5:** Adverse events and treatment compliance

Adverse events	Cisplatin + Etoposide compliance	Carboplatin + Etoposide compliance	p-value
Neutropenia	29 (61.7%)	19 (48.7%)	0.227
Anemia	27 (57.4%)	22 (56.4%)	0.923
Thrombocytopenia	20 (42.6%)	17 (43.6%)	0.923
Liver toxicity	3 (6.4%)	5 (12.8%)	0.459
Renal toxicity	8 (17%)	6 (15.4%)	0.838
Neutropenic fever	4 (8.7%)	6 (15.4%)	0.340
Dose reduction	8 (17%)	2 (5.1%)	0.087
Chemotherapy delay	12 (25.5%)	11 (28.2)	0.780
Chemotherapy termination	7 (14.9%)	7 (17.9%)	0.702

## Discussion

In this study, we investigated the demographic characteristics, therapeutic distributions, and laboratory and histological characteristics of extensive-stage SCLC cases in patients aged 65 years or older. We evaluated the relationship between these characteristics and OS and analyzed their prognostic values. We found that ECOG PS status was the most important negative prognostic factor for PFS and OS. There was no difference between carboplatin+etoposide and cisplatin+etoposide regimens in terms of PFS, OS, adverse events, and treatment compliance. Of note, the survival outcomes were consistent with the literature.

SCLC has a slower course in terms of therapeutic evaluation compared to other cancer types. Platinum+etoposide combination is still widely used in extensive-stage disease [1,6,7]. However, recently, the administration of anti-programmed death-ligand 1 agents such as atezolizumab and durvalumab together with platinum+etoposide combination in induction therapy and then continued as a maintenance treatment provided significant improvements in survival [8-10]. Our study consisted of a group receiving isolated cisplatin+etoposide or carboplatin+etoposide cytotoxic chemotherapy regimen. In a meta-analysis comparing cisplatin-based regimens with carboplatin-based regimens, no statistically significant difference was found in terms of OS, PFS, and objective response rate [11]. Similarly, in a phase 3 study comparing cisplatin+etoposide or carboplatin+etoposide in geriatric patients, most of whom were aged 70 years or older and with poor ECOG PS, response rates and survival rates were found to be similar [12]. In our study, in accordance with the literature, there was no difference in OS or PFS between the groups receiving cisplatin or carboplatin regimens. Although regimens with carboplatin have a better toxicity profile than regimens with cisplatin, in our study we found no difference in terms of adverse events and treatment compliance. Both regimens might be an option that can be preferred in the fragile geriatric age group. Furthermore, these results with geriatric SCLC patients were comparable with the younger ones with platinum+etoposide groups of pivotal trials [9]

In the study by Caprario et al., in which they evaluated approximately 10,000 patients diagnosed with SCLC aged 65 or older, approximately 65% of the patients received chemotherapy treatment. The median OS was nine months in patients receiving chemotherapy treatment [13]. Schild et al. evaluated approximately 145 patients aged 80 years or older with a diagnosis of SCLC. They found that the median OS of patients who received only chemotherapy treatment was 7.2 months [14]. Sundriyal et al. found a median OS of six months in geriatric patients with extensive-stage SCLC who received only chemotherapy treatment [15]. Similar to the above studies, we found the median OS as 8.2 months in our study. In addition, as in our study, the platinum+eoposide combination was preferred as the chemotherapy regimen in all three studies.

One of the most important factors affecting the treatment decision in extensive-stage SCLC is the patient's ECOG PS. The prognostic importance of ECOG PS has been shown in various studies involving all age groups [16-18]. There are limited studies on the isolated geriatric age group. In a study by Igawa et al., in which they evaluated the prognostic factors before second-line therapy in 731 extensive-stage SCLC patients aged 75 years or older, it was observed that longer survival was expected in patients with a good ECOG PS before first-line chemotherapy [19]. Again, Schild et al. showed that an ECOG PS of 0-1 was an independent prognostic factor for OS in their study in which they evaluated prognostic factors in patients with extensive stage SCLC aged 80 years or older [14]. In our study, we found that ECOG PS was an independent prognostic factor for both PFS and OS.

He et al. evaluated a total of 234 patients in their study, which included all age groups, and investigated the prognostic importance of LHD value before platinum-based treatment. They showed that high LDH before treatment was a negative prognostic factor for survival [20]. Hsieh et al. too showed that high LDH levels before treatment were an important prognostic marker of poor survival in their study involving approximately 1100 patients in all age groups [21]. In our study, while a high LDH value seen before treatment significantly affected both OS and PFS in univariate analysis, it lost its significance in multivariate analysis. This may be due to the relatively low number of patients in the current study compared to these studies.

The main limitations of our study are its retrospective design and the limited number of patients. In addition, although immunotherapy has proved its efficacy in first-line treatment of extensive-stage SCLC, all of our cases were treated with platinum+etoposide doublet regimen due to local reimbursement issues. Lastly, comorbid conditions and granulocyte colony-stimulating factor (GCSF) prophylaxis rates (primary or secondary) were not fully available.

## Conclusions

In our study, we revealed that ECOG PS was an independent prognostic factor for both PFS and OS in extensive-stage SCLC cases in the geriatric age group, which is a relatively marginalized population in the literature. In addition, we observed that objective response and disease control could be achieved in approximately half of the patients receiving chemotherapy. There was no difference between carboplatin+etoposide and cisplatin+etoposide regimens in terms of PFS, OS, adverse events, and treatment compliance. Furthermore, these results with geriatric SCLC patients were comparable with the younger ones with platinum+etoposide groups of pivotal trials. Thus, it may be an appropriate approach not to give up chemotherapy treatment easily in elderly patients with a diagnosis of extensive-stage SCLC. However, more prospective studies including the geriatric population are needed. It should be kept in mind that finding factors that might affect the prognosis and tailoring the treatment precisely on a case-by-case basis in geriatric cancer patients have an impact on survival.
